# The characteristics of and responses to the two COVID-19 outbreak waves in Hebei Province of China, January 2020 to February 2021

**DOI:** 10.1017/S0950268821002089

**Published:** 2021-09-17

**Authors:** Xiaomin Cheng, Yifan Li, Yali Zhang, Jiahai Lu

**Affiliations:** 1 School of Public Health, Sun Yat-sen University, Guangzhou, Guangdong Province510080, China; 2 One Health Center of Excellence for Research & Training, Sun Yat-Sen University, Guangzhou510080, China; 3 Key Laboratory for Tropical Disease Control of Ministry of Education, Sun Yat-Sen University, Guangzhou510080, China; 4 NMPA Key Laboratory for Quality Monitoring and Evaluation of Vaccines and Biological Products, Sun Yat-Sen University, Guangzhou510080, China

**Keywords:** COVID-19, Hebei Province, two outbreak waves

## Abstract

Hebei Province was affected by two coronavirus disease 2019 (COVID-19) outbreak waves during the period 22 January 2020 through 27 February 2020 (wave 1) and 2 January 2021 through 14 February 2021 (wave 2). To evaluate and compare the epidemiological characteristics, containment delay, cluster events and social activity, as well as non-pharmaceutical interventions of the two COVID-19 outbreak waves, we examined real-time update information on all COVID-19-confirmed cases from a publicly available database. Wave 1 was closely linked with the COVID-19 pandemic in Wuhan, whereas wave 2 was triggered, to a certain extent, by the increasing social activities such as weddings, multi-household gatherings and church events during the slack agricultural period. In wave 2, the epidemic spread undetected in the rural areas, and people living in the rural areas had a higher incidence rate than those living in the urban areas (5.3 *vs.* 22.0 per 1 000 000). Furthermore, *Rt* was greater than 1 in the early stage of the two outbreak waves, and decreased substantially after massive non-pharmaceutical interventions were implemented. In China's ‘new-normal’ situation, development of targeted and effective intervention remains key for COVID-19 control in consideration of the potential threat of new coronavirus strains.

## Background

Since the first coronavirus disease 2019 (COVID-19) case arising from infection with severe acute respiratory syndrome coronavirus 2 (SARS-CoV-2) was reported in China in December 2019, more than 174 million confirmed cases and 3.7 million deaths have been identified globally as of 9 June 2021. The initial wave of COVID-19 outbreaks in China has well abated in April 2020. However, since December 2020, several provinces in mainland China, such as Beijing, Liaoning, Hebei, Jilin and Heilongjiang, have experienced a new wave of infection triggered by travellers from overseas, or potentially contaminated imported frozen items. Although the ongoing massive vaccination provides the much-anticipated hope to end the COVID-19 pandemic, the rapidly emerging SARS-CoV-2 variants has posed a potential threat to the current arsenal of vaccines and monoclonal antibody therapies [1, 2]. Thus, in addition to the ongoing tracking of mutations and variants of SARS-CoV-2, continuous epidemiological investigations and the development of effective intervention are still crucial for pandemic control.

Hebei Province is located in North China, covering an area of 188 800 km^2^. The population of Hebei Province at the end of 2019 was 75.92 million, of which the urban population was 43.73 million and the rural population was 32.19 million. Hebei Province has experienced two heterogeneous waves of COVID-19 outbreaks from January 2020 to February 2021, with 318 confirmed cases in the first wave and 942 confirmed cases in the second wave. The clinical characteristics and treatment of critically ill patients with COVID-19 in Hebei Province during the first wave have been defined [3]. However, to the best of our knowledge, the epidemiology of the second wave, and comparisons of the characteristics and public health intervention between the two COVID-19 outbreak waves in Hebei Province have not yet been described. In this study, we assessed and compared the epidemiological characteristics, containment delay, cluster events and social activity, as well as non-pharmaceutical interventions of the two COVID-19 outbreak waves in Hebei Province. The findings will inform and help implement pandemic containment policies in the future.

## Methods

### Data source and variables’ definition

Real-time update information on all COVID-19-confirmed cases was collected from the official website of Health Commission of Hebei Province and the prefecture-level cities during periods of the first and second waves. The information of each confirmed case included age, gender, address, possible exposure, travel history and social activities within recent 14 days, and dates of symptom onset, diagnosis, isolation and hospital admission. Demographic information was obtained from the China Health Statistics Yearbook 2020.

We coded patients’ addresses into urban and rural areas according to the Zoning and Urban–Rural Division Code for Statistics in China in 2020. Clusters were defined as two or more patients with reported close contact [4]. Containment delay referred to the interval in days between symptom onset and isolation, quarantine camp, hospital or the first-positive viral RNA test of the patient. Wuhan/Hubei exposure history was identified as a history that patients travelled from/to Wuhan/Hubei within 1 month before onset [5, 6]. Patients or public involvement is not applicable and a full review by the Survey and Behavioural Research Ethics Committee was not required, because the study involves extraction of information from a publicly available database only.

### Statistical analysis

A descriptive analysis was performed, and epidemiological characteristics, containment delay, cluster events and social activity, as well as non-pharmaceutical interventions were compared between the first and second waves of COVID-19 outbreaks in Hebei Province. Categorical variables were reported as numbers and percentages whereas continuous variables were presented as median and interquartile ranges (IQRs). For continuous variables, we compared subgroups using the Mann–Whitney *U* test according to the distribution of datasets; for categorical variables, we compared subgroups using the chi-squared tests in SPSS 25.0. *P* < 0.05 was considered statistically significant. The spatial distribution of the confirmed cases in the two waves of COVID-19 outbreaks was mapped using ArcGIS software (version 10.7). The age/region-specific incidence analyses were based on the population of Hebei Province at the end of 2019. Graphics for containment delay were created using R (version 4.0.2). Network graphics for cluster events were created using Cytoscape software (version 3.7.2). Instantaneous reproduction number (*Rt*) based on daily confirmed cases was estimated over a 7-day sliding window with the EpiEstim R package. A Gamma distribution was applied for the generation time following a previous study, with mean generation time of 7.5 days and standard deviation of 3.4 days [7].

## Results

### Demographic and epidemiologic characteristics

**Fig. 1.**
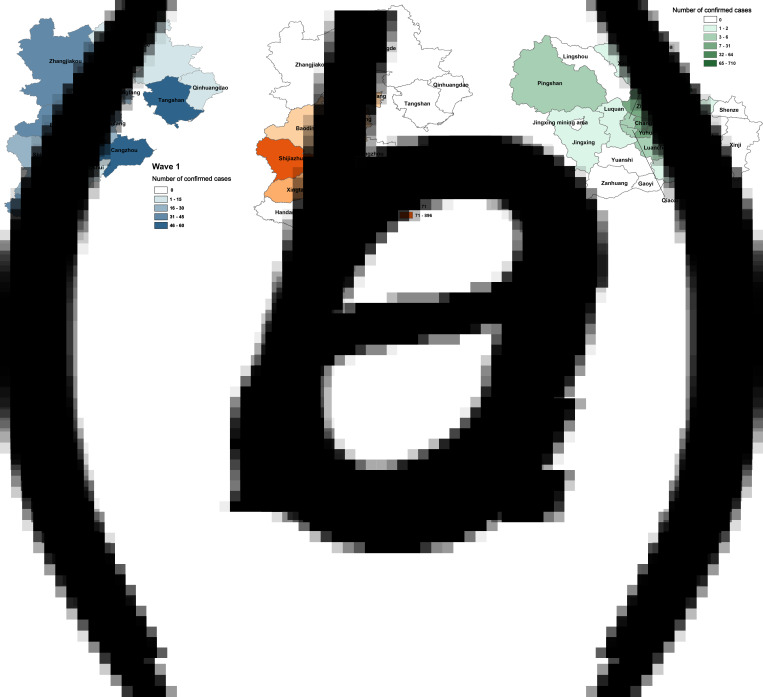
Spatial distribution of confirmed COVID-19 cases in Hebei Province. Number of confirmed cases in each prefecture-level city of Hebei Province (a) from 22 January 2020 to 27 February 2020 (wave 1), and (b) from 2 January 2021 to 14 February 2021 (wave 2). (c) Number of confirmed cases in each county-level administrative region of Shijiazhuang City.

**Fig. 2.**
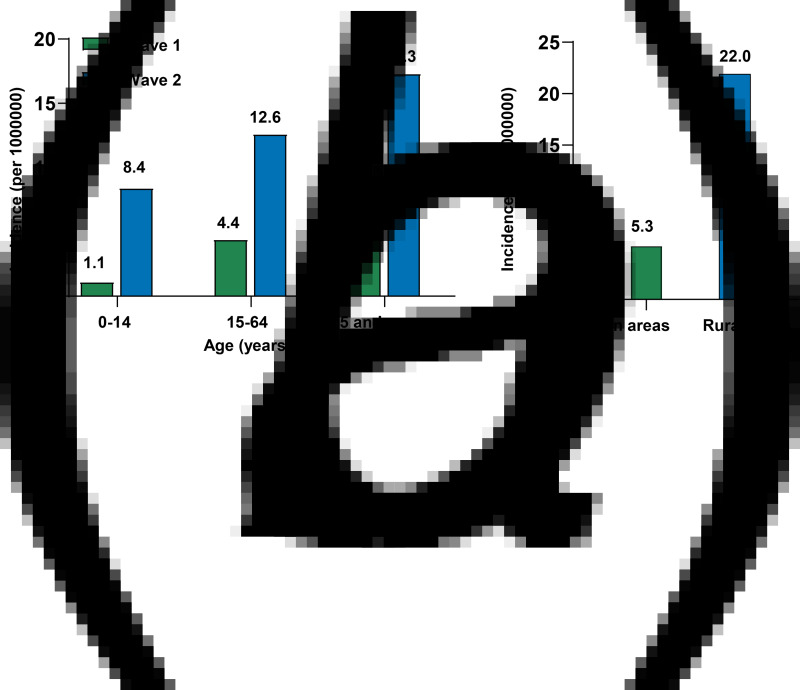
Cumulative incidence rates of COVID-19 in Hebei Province. (a) Cumulative age-specific incidence rates of COVID-19 in waves 1 and 2. (b) Cumulative region-specific incidence rates of COVID-19 in wave 2.

As depicted in Table 1, a total of 318 patients (median (IQR) age: 45.0 (32.0–57.8) years) were confirmed during the period 22 January 2020 through 27 February 2020 (wave 1), whereas 942 patients (median (IQR) age: 46.0 (30.0–60.0) years) were confirmed from 2 January 2021 to 14 February 2021 (wave 2). During wave 2, the proportion of confirmed cases reported in Shijiazhuang City increased significantly (*P* < 0.001), but the number of prefecture-level cities of Hebei Province reporting cases decreased (11 *vs.* 4, Figs 1a and b). Geographically, the distribution of cases was rather heterogeneous across the 22 county-level administrative districts of Shijiazhuang City during wave 2, with Gaocheng District recording the largest number of confirmed cases at 710 (Fig. 1c). As compared with wave 1, the proportion of female cases was significantly higher during wave 2 (48.1% *vs.* 58.7%, *P* = 0.001). A significant difference in age distribution of patients was observed between wave 1 and wave 2 (*P* < 0.001, Table 1). Moreover, an increased trend in the disease incidence with age was observed for both wave 1 and wave 2 (Fig. 2a). As compared with wave 1, COVID-19 incidence in wave 2 increased 7.6-, 2.9- and 3.6-fold in patients aged 0–14, 15–64 and >65 years, respectively. During wave 1, there were 85 cases with a Wuhan/Hubei exposure history and 70 of their close contacts. In wave 2, people living in the rural areas had a higher incidence rate than those living in the urban areas (5.3 *vs.* 22.0 per 1 000 000 person-years, Fig. 2b) and the percentage of rural and urban patients were 24.7% and 75.2%, respectively.

**Table 1.** Characteristics of the first and second waves of COVID-19 outbreak in Hebei Province

Variables	Wave 1 (*n* = 318)	Wave 2 (*n* = 942)	*P*
Time periods	22 January to 27 February 2020	2 January to 14 February 2021	
Case fatality rate, no. (%)	6/318 (1.9)	1/942 (0.1)	
Geographic			
City of Shijiazhuang	29/318 (9.1)	869/942 (92.3)	<0.001
Cities of Hebei Province affected^a^	11/11 (100.0)	4/11 (36.4)	
Sex, no. (%)			0.001
Male	165 (51.9)	389 (41.3)	
Female	153 (48.1)	552 (58.7)	
Median age (IQR), years	45 (32.0–57.8)	46 (30.0–60.0)	0.588
Age, years, no. (%)			<0.001
0–14	17 (5.4)	120 (12.7)	
15–64	238 (74.8)	648 (68.8)	
65 and over	49 (15.4)	172 (18.3)	
Unknown	14 (4.4)	2 (0.2)	
Wuhan-related exposure, no. (%)			
Wuhan/Hubei exposure history	85(26.7)	–	
Close contacts of case with Wuhan/Hubei exposure history	70(22.0)	–	
Residence, no. (%)			
Urban	–	233 (24.7)	
Rural	–	708 (75.2)	

a Number of prefecture-level cities with >1 reported case in a resident. There are 11 prefecture-level cities in Hebei Province.

### Containment delay

Among the 134 cases with a known date of symptom onset during wave 1, 67 cases were identified as having containment delay. In contrast, among the 98 cases with a known date of symptom onset during wave 2, 61 cases were regarded as having a containment delay. The median of containment delay during wave 1 was slightly longer than that of wave 2 but was not significantly different (median (IQR): 5.0 (2.0–7.0) days *vs.* 4.0 (2.0–7.0) days, *P* = 0.473).

**Fig. 3.**
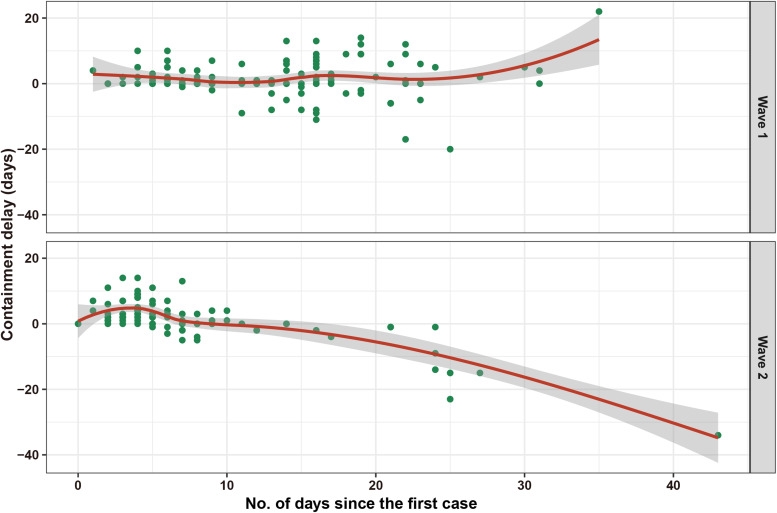
Containment delay of COVID-19 cases with a known date of symptom onset during the two waves of COVID-19 outbreak in Hebei Province. Days in abscissa indicates days between the diagnosis date of a certain case and the diagnosis date of the first cases (wave 1: 22 January 2020, wave 2: 2 January 2021).

To assess the containment delay before public health interventions implemented during wave 2, we calculated the containment delay for 53 cases whose symptoms onset occurring before reporting the first case (3 January 2021). Of those 53 symptomatic cases, 50 were regarded as having containment delay with a median delay of 4.0 days (IQR: 3.0–7.0). Besides, 49 of those 50 cases were rural residents, including 17 cases who did not seek any medical services when symptoms appeared, nine cases who self-medicated and 23 cases who visited primary care clinics in their villages. As indicated in Figure 3, since 10 days after the first case was identified, containment delay was no longer observed in the patients during wave 2. Specifically, cases have been identified mainly in isolation or active screening since then. In contrast, in wave 1, there were cases with containment delay even 20 days after laboratory confirmation of the first case.

### Cluster events and social activities

During wave 1, there were 53 clusters affecting a total of 224 patients (median (IQR) of clusters size: 3 (2–4) cases), wherein 35 clusters were related to at least one case who had a Wuhan/Hubei exposure history or who were close contacts of cases with Wuhan/Hubei exposure history (Supplementary Fig. S1). In addition, there were at least 43 clusters associated with household transmission. Specifically, the largest cluster, which occurred in Tangshan City, was made up of 25 cases and therein the earliest reported case was a shop assistant who had contact with people travelling from Wuhan. It was speculated that the second-largest cluster with a total of 14 cases was linked to travellers returning from Thailand, which occurred in Zhangjiakou City. The remaining clusters included 11 clusters involving more than five patients each, six clusters involving four patients each, 10 clusters involving three patients each and 24 clusters involving two patients each.

**Fig. 4.**
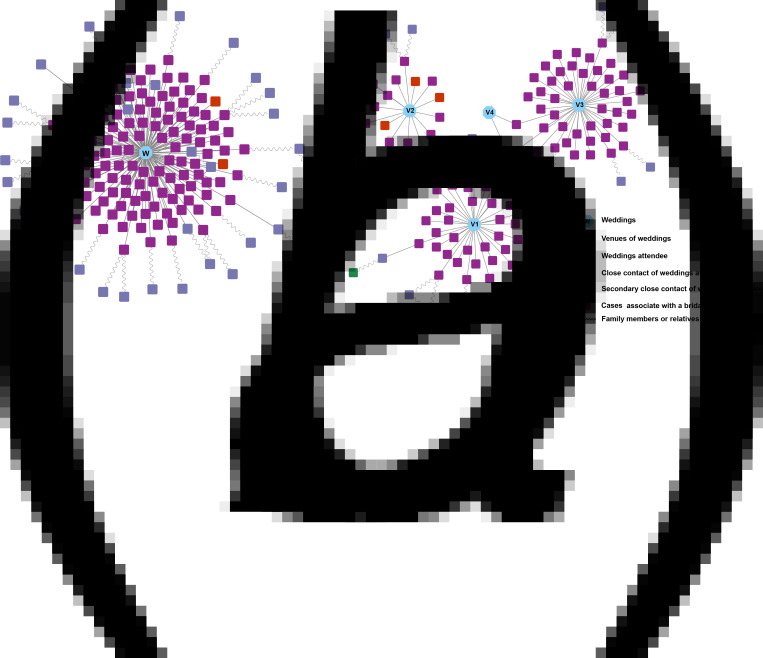
Cluster events associated with weddings in the second wave of COVID-19 outbreak. (a) Transmission network of the wedding-related cluster of undetermined source (*n* = 166). (b) The largest wedding-related cluster traced back to a collection of six wedding venues (*n* = 112).

During wave 2, the first case of COVID-19 was identified in Shijiazhuang City on 2 January 2021, who had a history of attending village religious gatherings, sporadic mask-wearing, visiting relatives and attending a wedding of about 250 people [8]. A total of 166 cases were associated with weddings at around 21 venues, including 128 wedding attendees, 33 close contacts of wedding attendees, two secondary close contacts of wedding attendees and three cases who went to a bridal shop at Xiaoguozhuang Village in Shijiazhuang City several times (Fig. 4a). Notably, the largest wedding-related cluster consisted of 112 cases and was traced back to a collection of six wedding venues in Shijiazhuang City from 28 December 2020 to 2 January 2021 (Fig. 3b). Among those 112 cases, five cases once went to two wedding venues and one case once went to three wedding venues (V1, V3 and V4). The remaining 91 wedding-unrelated clusters comprising a total of 208 cases involved two to six patients each, inclusive of 86 family-related clusters with a total of 200 cases.

The officers of Shijiazhuang City reported that there were 122 Christians in Xiaoguozhuang Village. Before the second wave of COVID-19 outbreak, some Christians gathered in a villager's house for religious activities. Moreover, epidemiological information showed that, within 14 days before the symptoms onset or the positive viral RNA test, at least 107 cases had a history of eating-out, 64 had a history of relatives visiting and 22 had a history of multi-household gatherings. Together, these social activities may have provoked the spread of the epidemic.

### Public health interventions

**Fig. 5.**
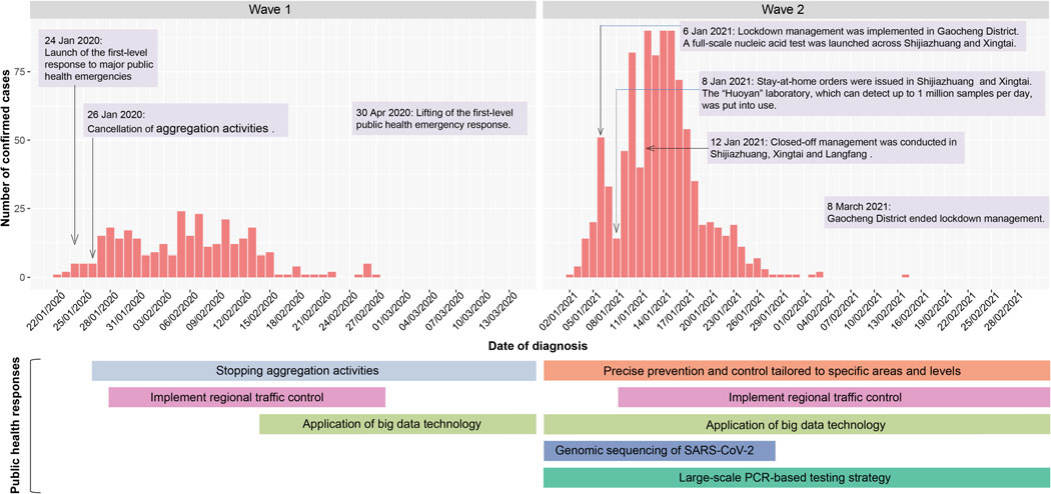
COVID-19 cases by date of diagnosis and non-pharmaceutical interventions taken to suppress COVID-19 transmission in Hebei Province.

**Fig. 6.**
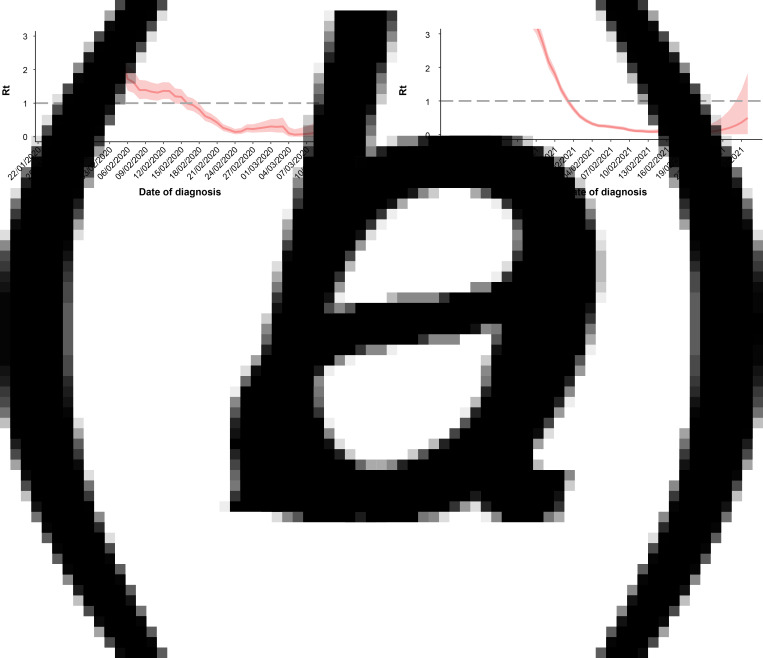
Effective reproduction number (*Rt*) for COVID-19 outbreak in Hebei Province during wave 1 (a) and wave 2 (b).

Increased social distancing and stringent restriction strategies such as cancellation of aggregation activities were adopted in both wave 1 and wave 2. Apart from the restriction of aggregation activities and regional traffic control, precise prevention and control tailored to specific areas and levels as well as large-scale polymerase chain reaction (PCR)-based testing strategy were implemented in wave 2 (Fig. 5). Depending on the demographic and epidemiological situation, epidemic risk level and adapted prevention and control strategies were assessed and determined for every county/district during wave 2. Particularly, lockdowns were implemented in the middle- or high-risk areas, where all personnel can only enter but no exit. Since 4 January 2021, lockdown management has been conducted in Zengcun Town of Gaocheng District, where the first case was identified. Two days later, as daily new cases grew quickly, lockdown management was expanded to all residential communities in Gaocheng District. Meanwhile, a full-scale PCR-based testing was launched across Shijiazhuang City and Xingtai City. In comparison with wave 1, the capacity for SARS-CoV-2 nucleic acid testing has been greatly boosted in wave 2. The ‘Huoyan’ nucleic acid test laboratory, which can detect up to 1 million samples per day, has put into use since 8 January 2021. During wave 2, a massive campaign of 49 million nucleic acid tests was conducted in Shijiazhuang City, including three rounds of nucleic acid detection in the whole city and 12 rounds of nucleic acid detection in high-risk areas such as Gaocheng District. As a result, a total of 631 positive samples were identified in the three rounds of nucleic acid detection across Shijiazhuang City. Notably, to find the source of infection in time, besides the application of big data technology in personal tracking, genomic sequencing of SARS-CoV-2 was implemented in the cases with the potential earliest onset date and the key cases with epidemiological association with the earlier cases in wave 2. *Rt* was greater than 1 in the early stage of COVID-19 outbreaks, implying a huge pressure of local transmission (Figs 6a and b). However, after massive non-pharmaceutical interventions were implemented, *Rt* plots showed substantially decreasing trends, suggesting that the interventions were effective at preventing the transmission of the disease.

## Discussion

In this study, we examined COVID-19 outbreaks that occurred in Hebei Province as two distinct waves from January 2020 to February 2021. The first wave was closely linked with the COVID-19 pandemic in Wuhan, as nearly half of the cases had a Wuhan/Hubei exposure history or had close contact with a person who had a Wuhan/Hubei exposure history. It was reported that the genomic characteristics of the Hebei strains in the second wave differed from recent strains detected in several COVID-19 epidemics in China and may have originated from the Russian strains [8]. In contrast to the first wave, the second wave was featured by a relatively limited geographic spread of COVID-19 but more confirmed cases.

Experience from the two COVID-19 outbreak waves in Hebei Province has proved that the decisive and strict non-pharmaceutical interventions made by Chinese government are remarkable. Despite the high total cost, non-pharmaceutical interventions including increased social distancing, stringent restriction and isolation strategies, precise prevention and control tailored to specific areas and levels, as well as active surveillance such as large-scale PCR-based testing strategies remains the over-riding public health priority to contain the spread of SARS-CoV-2 in the absence of effective therapies and herd immunity. The median of containment delay in the second wave was slightly shorter than that of the first wave. Unlike the first wave, containment delay was no longer observed in the second wave since 10 days after the first case was identified. This could be attributed to an elevated population awareness of the COVID-19 pandemic, behavioural modification in response to government measures and the boosted capacity for SARS-CoV-2 nucleic acid testing during the second wave [9]. A role for large-scale PCR-based testing strategies in epidemic control during the second wave should be highlighted, as it is conducive for the detection of presymptomatic and asymptomatic cases [10]. Comprehensive and active PCR testing strategies implemented for key populations and in outbreak settings have ensured timeliness of early case detection and containment of local outbreaks [11]. In addition, genomic sequencing of SARS-CoV-2 was performed during the second wave, which will help us to monitor the disease's spread and evolution of the virus. On the contrary, COVID-19 outbreak is a ‘big test’ that the second wave has exposed weaknesses in the public health system in Chinese rural areas. The second wave of COVID-19 outbreak started in rural areas, and rural residents had a higher COVID-19 incidence rate than urban residents (5.3 *vs.* 22.0 per 1 000 000 person-years). Notably, the epidemic spread undetected in Hebei Province until 2 January 2021, when the first case sought medical healthcare in a certain 3A-grade hospital and was then identified. On the subsequent days, 50 cases were identified to have symptom onset before that date, and therein 49 cases were rural residents. Among those 49 cases with a containment delay, 17 cases did not seek any medical services when symptoms appeared, nine cases self-medicated and 23 cases visited primary care clinics in their village. Together these findings suggest that weak awareness for healthcare and insufficient access to high-quality medical resources in rural residents have contributed to the second COVID-19 outbreak wave in Hebei Province to a certain extent. Primary care clinics failed to play their gatekeeper roles in disease triage as well as COVID-19 surveillance and monitoring in the early stage of the second wave, which is thought to be another important contributor to the containment delay in the second wave. Although the past few decades have seen great progress in rural health system capacity in China, there is still a large gap between rural and urban areas. Specifically, the majority of qualified health professionals and heavy medical equipment are concentrated in urban areas [12]. Based on the China Health Statistics Yearbook 2020, there were 11.10 health-care professionals per 1000 population in urban areas in comparison with 4.96 per 1000 population in rural areas in 2019. Based on statistics from 2019, 27.2% of country doctors in China were 60 years and above, and only 4.9% were under 35 years. Moreover, low education is especially common in country doctors [13]. The COVID-19 outbreak in Hebei Province has shown us that the rural areas have potential loopholes in pandemic prevention and control, both in terms of villagers’ awareness of disease prevention and the structure of current primary health care systems, and this should give the impetus for us to improve.

Both the first and second waves of COVID-19 outbreaks in Hebei Province occurred during the slack agricultural period in winter, which is consistent with previous studies wherein cold and dry environments in winter favour the survival and spread of SARS-CoV-2 [14–16]. Earlier studies have reported that community gatherings have the potential to be SARS-CoV-2 super-spreading events and might lead to the quick spread of COVID-19 in the community [17–19]. According to the epidemiological investigation and contact tracing records for cases, the second wave was triggered, at least in part, by the increasing social activities such as weddings, multi-household gatherings and church events during the slack agricultural period based on agriculture cropping patterns. Furthermore, previous studies have demonstrated that the effect of interventions may be gradually weakened over time due to public fatigue for social distancing as the pandemic continues [20, 21]. Concordance of these findings with ours suggests that public fatigue and decreased vigilance about COVID-19 might have given a boost to the larger second wave compared with the first wave in Hebei Province based on the cumulative number of confirmed cases.

This study has several limitations. First, details of patients’ addresses in the first wave were unavailable, so potential rural–urban disparities in COVID-19 incidence in the first wave and its comparison with the second wave could not be calculated. Second, reporting biases might exist in the epidemiological investigation and contact tracing records, which could result in inaccurate estimates of several variables such as possible exposure, travel history and social activities within recent 14 days. Third, the reliability of the estimation of *Rt* was limited by a delay between the onset date and diagnosis date of COVID-19 and a small number of confirmed cases.

## Conclusion

In summary, using the epidemiological data from the two COVID-19 outbreak waves in Hebei Province, our study suggests the lack of anti-epidemic awareness in rural areas is a lesson for other places to learn from. Despite the vaccine rollout, stopping the spread at the source and development of targeted and effective intervention remain key for COVID-19 control in consideration of the potential threat of new coronavirus strains.

## Data Availability

The data that support the findings of this study are openly available in the official website of Health Commission of Hebei Province (http://wsjkw.hebei.gov.cn/xxgzbdfyyqfk/index.jhtml) and the Zoning and Urban-Rural Division Code for Statistics in China in 2020 (http://www.stats.gov.cn/tjsj/tjbz/tjyqhdmhcxhfdm/2020/index.html).

## Supplementary material

For supplementary material accompanying this paper visit https://doi.org/10.1017/S0950268821002089. click here to view supplementary material
