# Supplementary figures and images for: The characteristics of and responses to the two COVID-19 outbreak waves in Hebei Province of China, January 2020 to February 2021
Source: Epidemiol Infect. 2021 Sep 17;149:e212. doi: 10.1017/S0950268821002089 (PMC8485049; doi:10.1017/S0950268821002089)

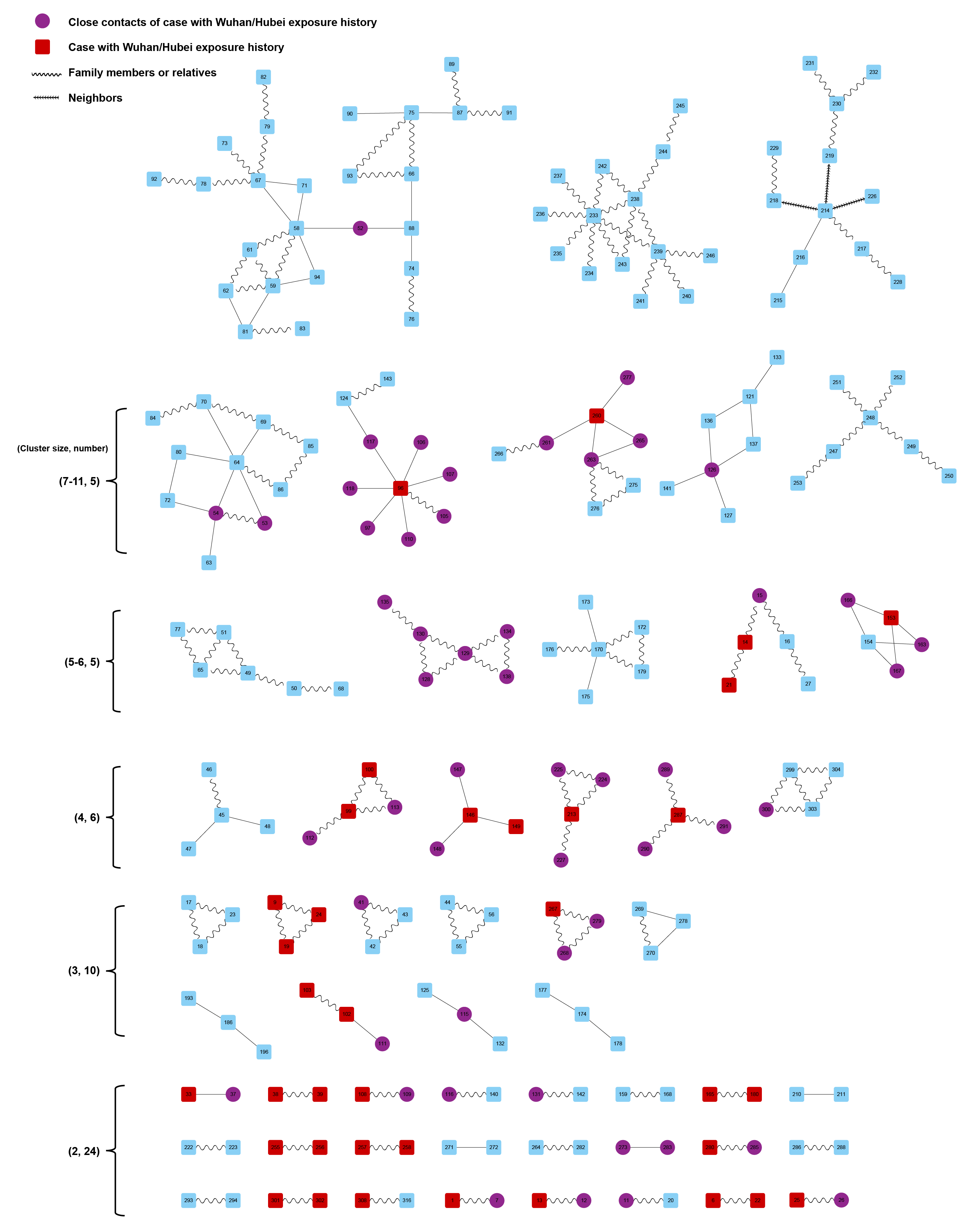

Supplement: Supplementary file 1 [file S0950268821002089sup001.png]
